# High-Pressure Synthesis and Structural Studies of La, Sm, Gd, and Dy Chlorides and Chloride Carbides

**DOI:** 10.1021/acsomega.5c09373

**Published:** 2026-01-09

**Authors:** Fariia Iasmin Akbar, Alena Aslandukova, Andrey Aslandukov, Yuqing Yin, Elena Bykova, Maxim Bykov, Dominique Laniel, Pavel Milkin, Timofey Fedotenko, Jonathan Wright, Anna Pakhomova, Gaston Garbarino, Mohamed Mezouar, Michael Hanfland, Natalia Dubrovinskaia, Leonid Dubrovinsky

**Affiliations:** † Institute of Inorganic and Analytical Chemistry, 425797 Goethe University Frankfurt, Max-von-Laue-Straße 7, Frankfurt 60438, Germany; ‡ Bavarian Research Institute of Experimental Geochemistry and Geophysics (BGI), 26523 University of Bayreuth, Universitätsstraße 30, Bayreuth 95440, Germany; § Material Physics and Technology at Extreme Conditions, Bavarian Research Institute of Experimental Geochemistry and Geophysics (BGI), 26523 University of Bayreuth, Universitätsstraße 30, Bayreuth 95440, Germany; ∥ Department of Physics, Chemistry and Biology (IFM), Linköping University, Linköping SE-581 83, Sweden; ⊥ Institute of Geosciences, 360318 Goethe University Frankfurt, Altenhöferallee 1, Frankfurt am Main 60438, Germany; # Centre for Science at Extreme Conditions and School of Physics and Astronomy, 97088 University of Edinburgh, Edinburgh EH9 3FD, United Kingdom; ∇ Faculty of Engineering Sciences, 332894 University of Bayreuth, Ludwig Thoma Str. 36A, Bayreuth 95447, Germany; ○ 28332 Deutsches Elektronen-Synchrotron DESY, Notkestrasse 85, Hamburg 22607, Germany; ◆ 55553 European Synchrotron Radiation Facility, CS 40220, Grenoble Cedex 9 38043, France

## Abstract

High-pressure synthesis provides unique pathways to materials with unprecedented structures and properties. Here we report the synthesis and structural characterization of novel rare-earth (La, Sm, Gd, Dy) chlorides, chloride carbides, and oxychloride phases obtained due to complex chemical reactions in diamond anvil cells after laser heating of rare-earth metals and NaCl at pressures of 39–127 GPa and temperatures of 2500–2800 K. Synchrotron single-crystal X-ray diffraction analysis allowed us to solve previously unknown crystal structures of binary (La_2_Cl, LaCl, LaCl_3_, DyCl) and ternary (DyNa_2_Cl_5_, Sm_2_ClC_2_, Gd_2_ClC_2_, Dy_2_ClC_2_, Sm_19_ClC_18_, Gd_19_ClC_18_, Dy_5_Cl_3_C, DyOCl) compounds. Significantly, we identified *trans*-polyacetylene-like carbon chains in lanthanide chloride carbides, a structural motif previously hypothesized but not observed experimentally. Our findings highlight the enhanced chemical reactivity of alkali halides under extreme conditions, uncovering novel chemical bonding and expanding the landscape of potential functional materials accessible through high-pressure synthesis.

## Graphical abstract

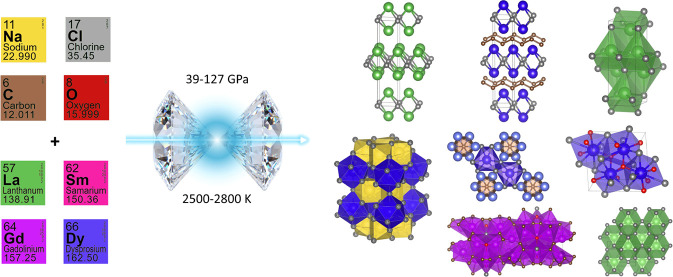

## Introduction

Rare-earth element (REE) chlorides and chloride carbides are of considerable interest in science and technology due to their distinctive physical and chemical properties, which enable a wide range of applications. REE chlorides serve as essential precursors in materials synthesis,^1^ metallurgy,^2^ and catalysis,^3^ while chloride carbides—though less explored—exhibit intriguing structural diversity and potential for advanced functional materials.^4,5^ Their unique electronic,^6^ magnetic,^7^ and optical properties make them valuable in fields such as optics,^8^ energy storage,^9,10^ and catalysis.^11,12^ Understanding and harnessing these compounds can lead to advancements in cutting-edge technologies, including superconductors, fuel cells, and environmental remediation strategies.

REE chlorides and chloride carbides can be synthesized by several established methods. Direct chlorination and carbochlorination of REE_2_O_3_ with chlorine gas at high temperatures (in the presence of carbon in the case of carbochlorination) yield anhydrous REECl_3_.^13^ Chloride carbides (REE–Cl–C) are typically obtained via solid-state reactions involving REEs, graphite, and REE trichlorides (REECl_3_) under an inert atmosphere at high temperatures.^14–16^ Another example is hydrothermal synthesis, which is particularly useful for creating layered and high-entropy REE compounds with controlled stoichiometry and crystal structures.^17^ While these methods are well-known, high-pressure synthesis in laser-heated diamond anvil cells (DACs) using Cl-bearing precursors remains an emerging approach, offering the potential for novel REE phases with unique properties.

In the present work, we report the synthesis and structural studies of chlorides, chloride carbides, and oxychlorides of La, Sm, Gd, and Dy, obtained due to chemical reactions of REEs with NaCl during laser heating in DACs at pressures of 39 to 127 GPa. All compounds were analyzed *in situ* at high pressures by using synchrotron single-crystal X-ray diffraction (SCXRD). Crystal structures of many previously unknown binary (*tI*6 La_2_Cl, *oC*8 LaCl, *oP*8 LaCl_3_, and *cP*2 DyCl) and ternary compounds (*oI*32 DyNa_2_Cl_5_, *oC*10 Sm_2_ClC_2_, *oC*10 Gd_2_ClC_2_, *oC*10 Dy_2_ClC_2_, *mP*76 Sm_19_ClC_18_, *mP*76 Gd_19_ClC_18_, *hP*18 Dy_5_Cl_3_C, and *oP*12 DyOCl) have been solved and refined. Our work contributes to the development of synthesis methods for lanthanide chlorides and chloride carbides, extending knowledge of their crystal structures and crystal chemistry.

## Materials and Methods

### Sample Preparation

In our experiments, we utilized BX90-type DACs with a large X-ray aperture.^18^ We employed Boehler-Almax-type diamonds as anvils with culet diameters of 80/120/250 μm. Rhenium gaskets with an initial thickness of 200 μm were indented to ∼16/22/28 μm, and a hole of ∼35/55/105 μm in diameter was laser-drilled in the center of the indentation. Thin pieces of metals (Sc, Y, La, Sm, Gd, or Dy; 99.9%, ChemPur or smart-elements) were placed directly on the diamond anvil (Figure 1a) and covered by dry sodium chloride, NaCl (99.999% purity, ChemPUR) which served as a thermal insulator, pressure-transmitting medium, and reactant. Contact with the diamond anvil enabled a carbon source for chemical reactions with the metals. There is another way to place the metal in the DAC so that it is isolated from the anvils by the pressure-transmitting medium (Figure 1b). Although we did not use this configuration, we discuss below how the different arrangements affect the experimental results.

**Figure 1:**
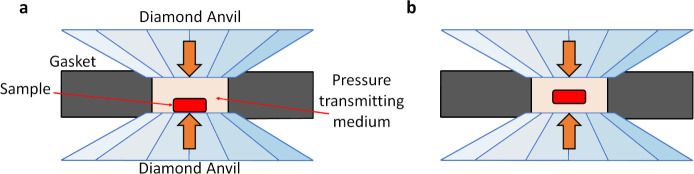
A schematic representation of the cross-section of the pressure chamber showing different sample loading geometries. (a) Thin pieces of a metal are placed directly on a diamond anvil (used in the current study). (b) The metal is isolated from the anvils by the pressure medium.

Samples were compressed to the desired pressure and laser-heated at 2500(200)–2800(200) K using an *in-house* double-sided YAG laser-heating setup^19^ (1064 nm wavelength). Thermal emission spectra from the heated area were collected using an IsoPlane SCT 320 spectrometer with a 1024 × 2560 PI-MAX 4 camera.^19^ The pressure was determined using the NaCl equation of state (EoS).^20,21^

### X-ray Diffraction

The reaction products were analyzed by synchrotron single-crystal X-ray diffraction (SCXRD) measurements at the P02.2 beamline (λ = 0.2904 Å, beam size ∼ 1.5 × 2 μm^2^) of Deutsches Elektronen-Synchrotron (DESY, PETRA III, Hamburg, Germany) and at three synchrotron beamlines of the European Synchrotron Radiation Facility (ESRF, Grenoble, France): ID11 (λ = 0.2846 Å, beam size ∼ 0.75 × 0.75 μm^2^), ID15B (λ = 0.4100 Å, beam size ∼ 1.5 × 2 μm^2^), and ID27 (λ = 0.3738 Å, beam size ∼ 2 × 2 μm^2^). At the P02.2 beamline of DESY, diffraction patterns were collected on a PerkinElmer 1621 XRD flat-panel detector. At the ID11 beamline of the ESRF, data acquisition was performed with an Eiger2X CdTe 4 M hybrid photon-counting pixel detector, while at ID15B and ID27, an Eiger2X CdTe 9 M hybrid photon-counting pixel detector was used. Powder X-ray diffraction (PXRD) images were collected upon continuous sample rotation in a range of ±1° around the vertical ω axis. During single-crystal data collection, the cell was rotated around the vertical ω axis from −38° to +38° with narrow 0.5° steps. Creating maps with XDI software^22^ helps to visualize the phase distribution within the pressure chamber and to locate areas where the SCXRD data collections should be performed. The CrysAlis^Pro^ software package^23^ was used for the analysis of the SCXRD data (peak hunting, indexing, data integration, frame scaling, and absorption correction). To calibrate an instrument model in the CrysAlis^Pro^ software, i.e., the sample-to-detector distance, the detector’s origin, offsets of the goniometer angles, and rotation of both the X-ray beam and the detector around the instrument axis, we used a single crystal of orthoenstatite [(Mg_1.93_,Fe_0.06_)­(Si_1.93_,Al_0.06_)­O_6_, *Pbca* space group, *a* = 8.8117(2) Å, *b* = 5.18320(10) Å, and *c* = 18.2391(3) Å]. The DAFi program was used for the search of reflection groups belonging to the individual single-crystal domains.^24^ Using the OLEX2 software package,^25^ the structures were solved with the ShelXT structure solution program^26^ using intrinsic phasing and refined with the ShelXL^27^ refinement package using least-squares minimization. Crystal structure visualization was made with VESTA software.^28^ The polyhedral assignments were made according to the fitting results obtained using Polynator software.^29^

## Results and Discussion

The experimental details are summarized in Table S1 (Supporting Information). In addition to chlorides, chloride carbides, and oxychloride (the latter formed due to partial oxidation of Dy during sample loading), we observed the formation of numerous carbides. The results of the carbide studies will be published separately.

In this paper, we describe all newly identified chlorine-containing phases, beginning with binary and ternary chlorides, followed by chloride carbides—ordered by increasing chlorine content per formula unit—and concluding with an oxychloride. A general discussion follows the phase descriptions.

### Structures of Novel Lanthanide Chlorides

#### Lanthanum Chlorides

In the DAC loaded with La and NaCl, three lanthanum chloride phases were detected as products of chemical reactions (Tables S1 and S2).

The previously unknown *tI*6 La_2_Cl phase, discovered at 81(2) GPa, has a tetragonal unit cell (space group *I*4/*mmm*, #139, *Z* = 2, Table S2), in which four lanthanum atoms occupy the 4*e* Wyckoff site and two chlorine atoms occupy the 2*a* positions (Figure 2a, Table S2). In the structure of *tI*6 La_2_Cl (Figure 2a) (anti-LaI_2_ structure,^30^ MoSi_2_ structure type^31^ (CuZr_2_-type according to the ICSD database^32^)), each La atom is connected to four Cl atoms at a distance of 2.7579(12) Å. In a simple geometrical representation of the structure, the La_2_Cl_4_ groups form octahedra, and these octahedra are connected through Cl–Cl edges to form layers. The layers are packed in an ABAB··· sequence and are shifted by 1/2 of the diagonal of the octahedra relative to each other. To the best of our knowledge, Y_2_Cl is the only other rare-earth chloride with a REE_2_Cl stoichiometry reported to date, having been both theoretically predicted^33^ and experimentally synthesized.^4^ Despite their identical stoichiometries, Y_2_Cl and La_2_Cl adopt different crystal structures.

**Figure 2:**
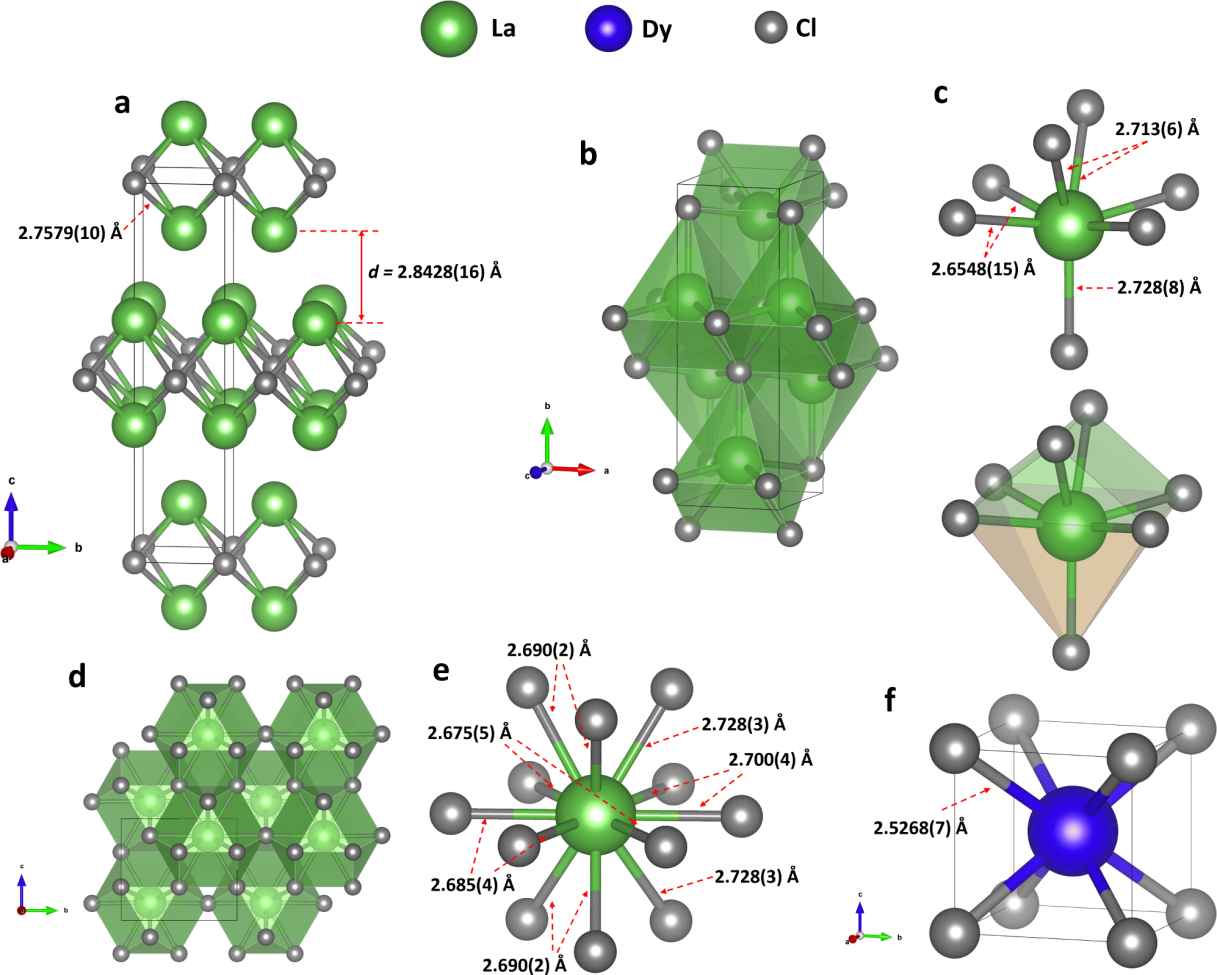
Crystal structures of the newly synthesized lanthanum and dysprosium chlorides. (a) *tI*6 La_2_Cl at 81(2) GPa, stick-and-ball model. (b) *oC*8 LaCl at 80(2) GPa, polyhedral model; (c) coordination of La atoms with interatomic distances (top) and highlighted polyhedron shape (bottom). (d) *oP*8 LaCl_3_ at 81(2) GPa, polyhedral model viewed along the *a* direction; (e) coordination of La atoms. (f) *cP*2 DyCl at 95(3) GPa, a unit cell. Green, gray, and blue spheres represent lanthanum, chlorine, and dysprosium atoms, respectively; unit cells are outlined by thin black lines.

The lanthanum monochloride *oC*8 LaCl (space group *Cmcm*, #63, *Z* = 4, Table S2) synthesized at 80(2) GPa has the TlI structure type (B33).^34^ The previously known *hR*12 LaCl polymorph^35^ has the ZrCl-type structure (space group *R*-3*m*, #166, *Z* = 6). The *oC*8 LaCl orthorhombic unit cell contains lanthanum and chlorine atoms occupying Wyckoff site 4*c* (Figure 2b). Lanthanum atoms are coordinated by seven chlorine atoms at distances of either 2.6548(15), 2.713(6), or 2.728(8) Å in the capped trigonal prismatic molecular geometry, forming a kind of an augmented triangular prism (or monocapped isosceles wedge, according to the Polynator fitting^29^)—a polyhedron constructed by attaching a pyramid onto the rectangular face of a triangular prism (shown in orange and green, respectively) (Figure 2c). The apexes of the prisms are alternately oriented in two opposite directions along the *b*-axis as shown in Figure 2b; the trigonal prisms share faces, whereas the pyramids share edges.

The lanthanum trichloride *oP*8 LaCl_3_ crystallized at 81(2) GPa. Its orthorhombic structure (space group *Pmmn*, #59, *Z* = 2) has not been observed previously. The other known polymorph, *hP*2 LaCl_3_,^36^ has the UCl_3_-type structure (space group *P*6_3_/*m*, #176, *Z* = 2). In the structure of *oP*8 LaCl_3_ (isostructural to LaF_3_, anti-Cu_3_Ti structure type^37^ (β–TiCu_3_-type according to the ICSD database^32^)), lanthanum atoms occupy a single 2*a* Wyckoff position. They are coordinated by twelve chlorine atoms (on the 4*e* (Cl1) and 2*b* (Cl2) Wyckoff sites) at distances of 2.675(5)-2.728(3) Å (Figure 2d, e, Table S2), forming an anticuboctahedron polyhedron—composed of eight triangular and six square faces. The polyhedra form columns along the *b* direction through corner sharing. The columns are interconnected through sharing of triangular and square faces.

### Dysprosium Chlorides

The dysprosium monochloride, *cP*2 DyCl, was synthesized at pressures of 95(3) and 121(3) GPa, differing from the previously discovered *hP*4 DyCl at 40 GPa^4^ (NiAs-type structure, space group *P*6_3_/*mmc*, #194, *Z* = 2). This novel compound has a CsCl-type structure (space group *Pm*-3*m*, #221, *Z* = 1), with chlorine and metal atoms occupying 1*a* and 1*b* Wyckoff positions, respectively (Table S3). Dysprosium atoms are coordinated by the eight nearest chlorine atoms with a Dy–Cl distance of 2.5268(7) Å at 95(3) GPa (Figure 2f).

The novel dysprosium–sodium chloride *oI*32 DyNa_2_Cl_5_ (*I*4/*mcm*, #140, *Z* = 4) with the NH_4_Pb_2_Br_5_-type structure^38–40^ (RbPb_2_Br_5_-type according to the ICSD database^32^) was observed at 107(3) GPa (Figure 3, Table S4) among the products of high-pressure high-temperature (HPHT) reaction between Dy and NaCl. The Dy, Cl1, Cl2, and Na atoms occupy the 4*a*, 16*l*, 4*c*, and 8*h* Wyckoff sites, respectively. Dysprosium atoms are coordinated by ten chlorine atoms at distances of *d*(Dy1–Cl1) = 2.4635(9) Å and *d*(Dy1–Cl2) = 2.550(3) Å at the synthesis pressure (Figure 3c), forming a bicapped tetragonal antiprism. Sodium atoms are in a bicapped trigonal prism formed by eight chlorine atoms with interatomic distances of *d*(Na1–Cl1) = 2.2792(15) or 2.3208(15) Å and *d*(Na1–Cl2) = 2.2913(13) Å (Figure 3d). The phases with the NH_4_Pb_2_Br_5_-type structure are described in the literature^38–40^ by the general formula Me1^+^(Me2^2+^)_2_X^–^, where Me1 and Me2 are electropositive, and X is an electronegative atom. In *oI*32 DyNa_2_Cl_5_, Me1 = Dy^3+^ and Me2 = Na^+^ which makes it distinct from previously known compounds.

**Figure 3:**
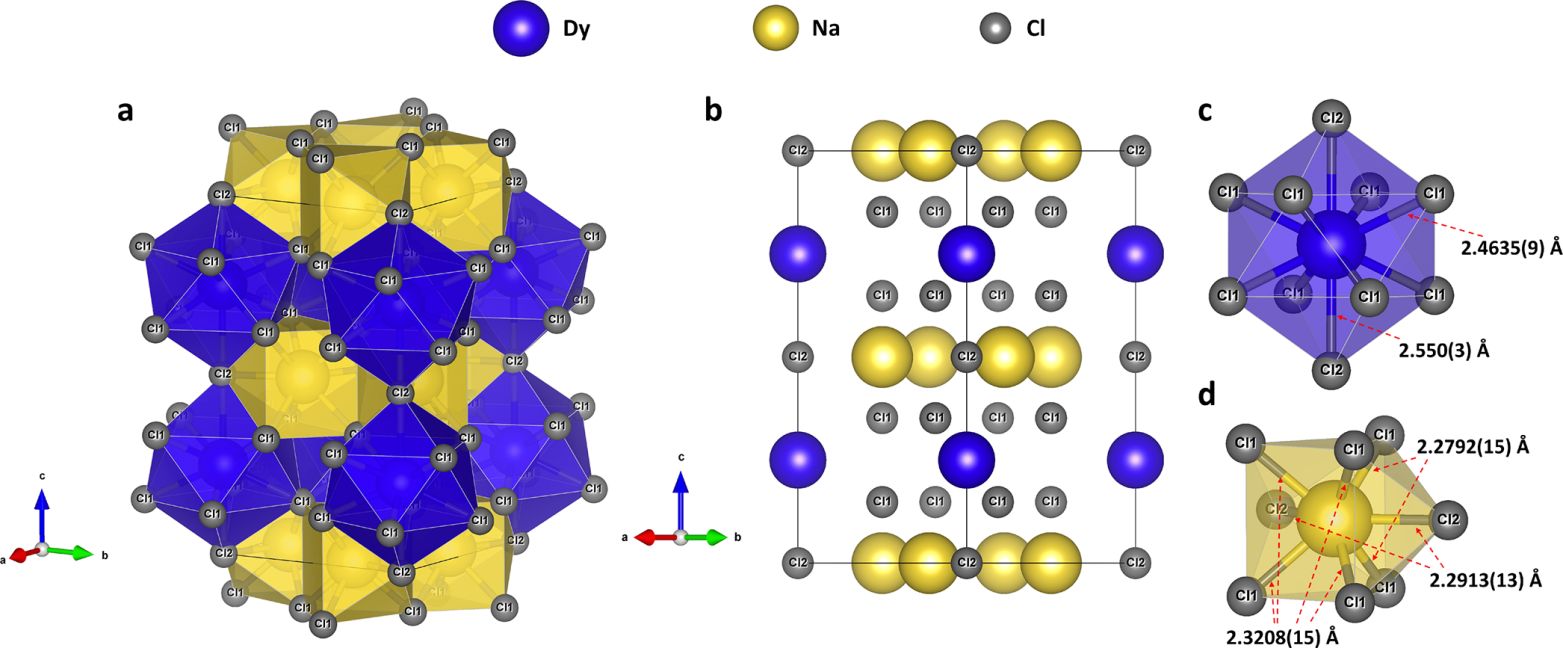
Crystal structure of *oI*32 DyNa_2_Cl_5_ at 107(3) GPa. (a) A polyhedral model. (b) A ball model viewed along the [110] direction. Coordination of the (c) Dy and (d) Na atoms. Dysprosium—blue, sodium—yellow, and chlorine—gray.

### Structures of Rare-Earth Metal Chloride Carbides

#### Chloride Carbides with the Mn_2_AlB_2_-Type Structure

Three rare-earth ternary compounds, *oC*10 REE_2_ClC_2_ (REE = Sm, Dy, Gd), synthesized in this work, exhibit the Mn_2_AlB_2_-type structure (space group *Cmmm*, #65, *Z* = 2) previously known mainly for borides,^41,42^ but unknown for carbides and chlorides. Their orthorhombic unit cells contain REE, chlorine, and carbon atoms located at the 4*j*, 2*a*, and 4*i* Wyckoff sites, respectively (Figure 4a, Table S5). Carbon atoms form *trans*-polyacetylene-like chains along the *a*-axis with a C–C distance of 1.549(14) Å and an angle ∠(C–C–C) = 123.9(19)°.

**Figure 4:**
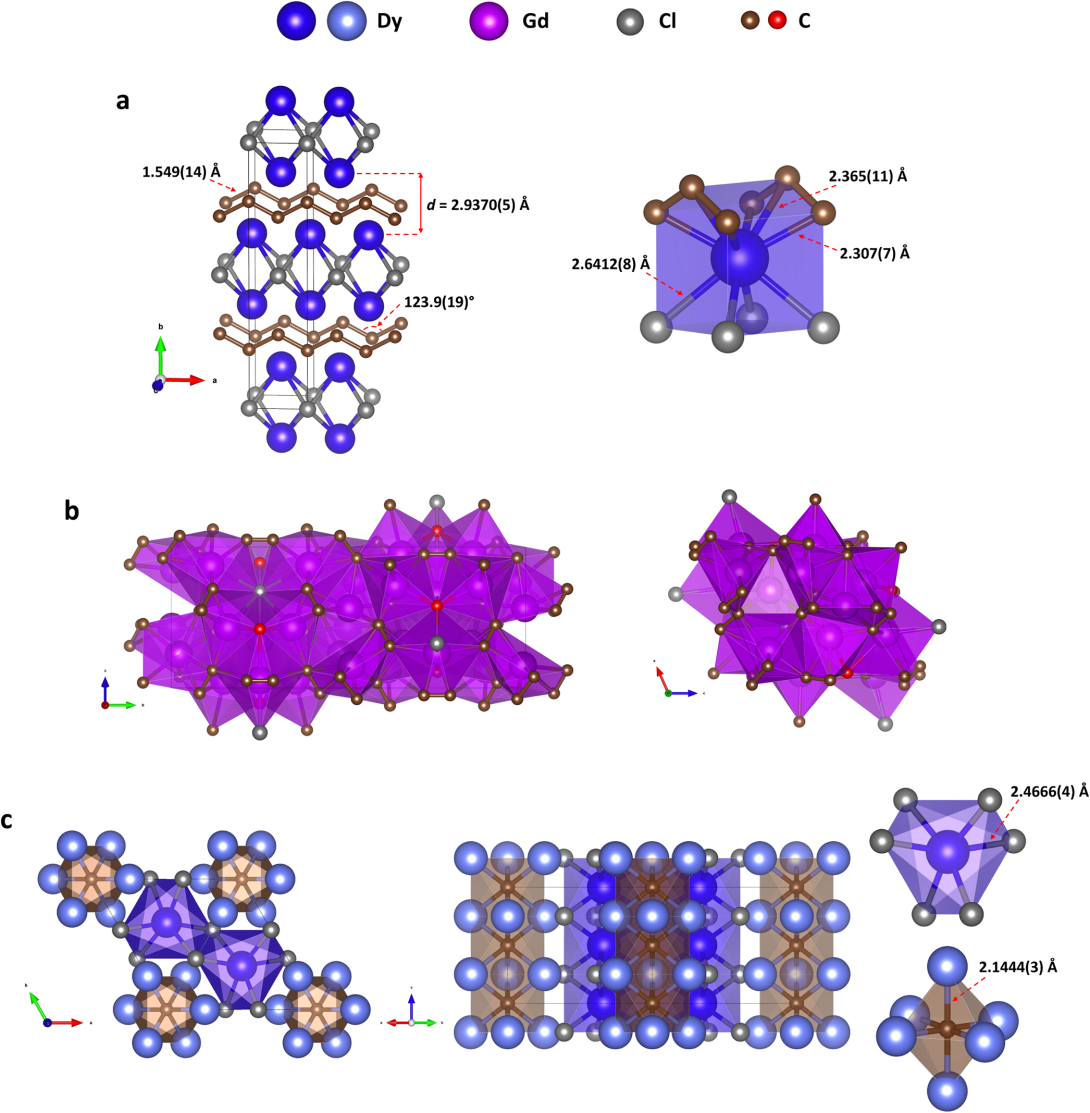
Crystal structures of rare-earth metal chloride carbides *oC*10 Dy_2_ClC_2_ (and isostructural Gd_2_ClC_2_ and Sm_2_ClC_2_), *mP*76 Gd_19_ClC_18_ (and isostructural Sm_19_ClC_18_), and *hP*18 Dy_5_Cl_3_C. (a) *oC*10 Dy_2_ClC_2_ at 76(3) GPa: stick-and-ball model (left) and coordination of Dy atoms (right). (b) *mP*76 Gd_19_ClC_18_ at 45(2) GPa: polyhedral model as viewed along the *a* (left) and *b* (right) directions. (c) *hP*18 Dy_5_Cl_3_C at 122(3) GPa; from left to right: polyhedral model viewed along the *c* axis and [110] direction; C­(Dy2)_6_ octahedron; Dy1Cl_6_ twisted trigonal prism. Dysprosium atoms in two crystallographically distinct positions are shown with different colors: Dy1—dark blue, Dy2—light blue; Gd—purple; Cl—gray; C catenated—brown; discrete C atoms—red.

#### Chloride Carbides with Sm_19_ClC_18_-Type Structure

The rare-earth chloride carbides *mP*76 Sm_19_Cl_0.81_C_18_ and *mP*76 Gd_19_ClC_18_ (*P*2_1_/*m*, #11, *Z* = 2) were synthesized at pressures of 39(2) and 45(2) GPa, respectively. The unit cell of Gd_19_ClC_18_ comprises 11 crystallographically distinct metal atoms (8 of them at the 4*f* and three at the 2*e* Wyckoff positions), 10 distinct carbon atoms (8 at the 4*f* site form dumbbells, whereas the other two at the 2*e* site are not catenated), and one chlorine atom at the 2*e* Wyckoff site (Table S6). Metal atoms are surrounded by carbon and chlorine atoms forming various irregular polyhedra with coordination numbers of 6, 7, or 8. Figure 4b presents the polyhedral model, as viewed along the *a* (left) and *b* (right) directions. The interatomic distances are presented in Supplementary Table S6. The structure of the samarium compound exhibits a pronounced residual electron density peak located 0.704(9) Å from Sm11, which may be attributed to positional disorder of the samarium atom. In the initial refinement, the model displayed a chemically unreasonable Sm12–Cl1 distance and a relatively high atomic displacement parameter (ADP) for the chlorine atom. Subsequent refinements of the occupancies for the Sm11, Sm12, and Cl1 sites markedly improved the structural model (Table S6). The results indicate that, in a part of the structure, samarium occupies the Sm11 position with chlorine present at the Cl1 site, whereas in the remaining fraction, samarium is located at the Sm12 position with no chlorine in its first coordination sphere.

The novel samarium and gadolinium chloride carbides contain carbon in the form of discrete atoms and dumbbells. Assuming that the metal atoms have an oxidation state of 3+, the charge distribution can be represented as REE^3+^_19_Cl^–^[C_2_]^6–^_8_C^4–^_2_. The assumed formal charges suggest a single-bond character in the [C_2_] dumbbells.

#### Dysprosium Chloride Carbide

The novel dysprosium chloride carbide *hP*18 Dy_5_Cl_3_C crystallized at 122(3) GPa. Having a hexagonal symmetry (space group *P*6_3_/*mcm*, #193, *Z* = 2) (Figure 4c, Table S7), it adopts the Cr_5_Si_3_B-type^43^ structure known for B-, C-, N-, and O-containing ternary compounds M_5_X_3_L_*x*_ (L = B, C, N, O, Cl, Br, or I, *x* ≤ 1), where M is a metal (REE, Ti, Zr, Hf, V, Nb, Ta, Cr, Mo, or Mn) and X is typically Zn, Cd, Al, Ga, In, Tl, Si, Ge, Ga, Sn, As, Sb, or Bi.^44–52^ This structure type is a derivative of the Ga_4_Ti_5_ structure type (the CCDC deposition number 103997).^53^ The latter was recently reported for novel isostructural sodium polychloride *hP*18-Na_4_Cl_5_ and sodium polybromide *hP*18-Na_4_Br_5_.^54^

In the structure of *hP*18 Dy_5_Cl_3_C, C and Cl are at the 2*b* and 6*g* Wyckoff sites, and Dy1 and Dy2 are at the 4*d* and 6*g*, respectively. Geometrically, Dy1 atoms form linear chains aligned along the *c* direction with a Dy1–Dy1 distance of 2.4188(7) Å. C atoms also form linear chains along the *c* direction with a C–C distance of 2.4188(7) Å (Figure 4c). C atoms are coordinated by six Dy2 atoms, forming an octahedron with an edge length of 2.9978(5) or 3.0672(3) Å. Dy1 atoms have a twisted trigonal prismatic coordination by the six nearest equidistant chlorine atoms with *d*(Dy1–Cl1) = 2.4666(4) Å (at the synthesis pressure). Thus, the structure of *hP*18 Dy_5_Cl_3_C is easily visualized as being formed by two distinct columnar arrangements in the *c* direction, those of face-sharing C­(Dy2)_6_ octahedra and face-sharing Dy1Cl_6_ twisted trigonal prism (see the polyhedral model viewed along the *c* and [110] directions in Figure 4c).

#### Structure of Dysprosium Oxychloride

The previously unknown modification of dysprosium oxide chloride *oP*12 DyOCl (space group *Pnma*, #62, *Z* = 4) (Figure 5, Table S8) with the TiNiSi-type^55^ (or SrMgSi-type^56^) structure was synthesized in the current work at 76(3) and 108(3) GPa. The novel compound differs from the previously reported *tP*6 DyOCl^57,58^ (PbFCl-type structure, space group *P*4/*nmm*, #129, *Z* = 2). In the structure of *oP*12 DyOCl, dysprosium, oxygen, and chlorine atoms occupy the Wyckoff position 4*c*. Four oxygen and six chlorine atoms coordinate Dy atoms forming a bicapped tetragonal antiprism, which share both triangular and square faces (Figure 5b, c). The interatomic distances at 108(3) GPa are *d*(Dy–O) = 2.105(4)–2.112(4) Å and *d*(Dy–Cl) = 2.4280(12)–2.9279(13) Å (Figure 5c).

**Figure 5:**
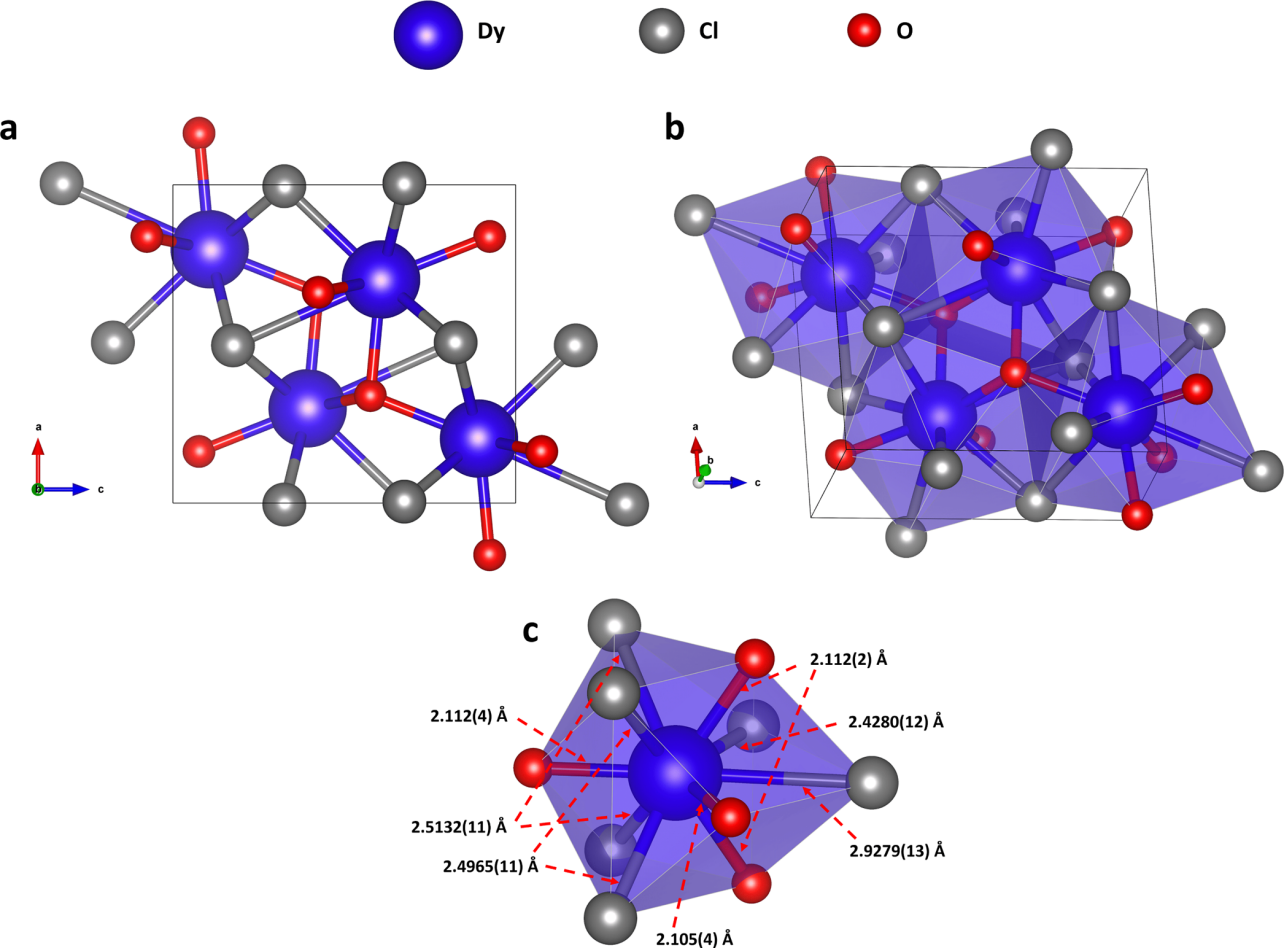
Crystal structure of *oP*12 DyOCl at 108(3) GPa. (a) Stick-and-ball model projected along the *a* axis. (b) Perspective view of the polyhedral model. (c) Coordination of Dy atoms. Dysprosium atoms are blue, oxygen is red, and chlorine is gray.

## Discussion

Alkali halides, especially sodium chloride, are known for their chemical inertness in the solid form. Due to this, in HPHT experiments, they have been used as inert pressure-transmitting media,^59^ electrical and thermal insulators,^59–61^ and pressure calibrants.^20,62^ However, recent advances in both experimental and theoretical investigations have shed light on the unusual behavior of the Na–Cl system under high pressure, revealing the formation of several compounds with atypical stoichiometry, such as Na_4_Cl_5_ and Na_2_Cl_3_,^54^ for example. It was found that NaCl used as a pressure-transmitting medium in experiments with REEs reacts not only with metals but also with carbon from diamond anvils, leading to the formation of not only metal chlorides, such as Y_2_Cl and DyCl, but also chloride carbides, Y_2_ClC and Dy_2_ClC.^4^

In the course of this work, we have demonstrated that the synthesis of new binary and ternary Cl-bearing compounds from metals and NaCl (plus C and O) can be carried out deliberately and reproducibly. Twelve new compounds have been synthesized: *tI*6 La_2_Cl, *oC*8 LaCl, *oP*8 LaCl_3_, *cP*2 DyCl, *oI*32 DyNa_2_Cl_5_, *oC*10 Sm_2_ClC_2_, *oC*10 Gd_2_ClC_2_, *oC*10 Dy_2_ClC_2_, *mP*76 Sm_19_ClC_18_, *mP*76 Gd_19_ClC_18_, *hP*18 Dy_5_Cl_3_C, and *oP*12 DyOCl. All of them, except one (*mP*76 Sm_19_ClC_18_), crystallize in known structural types (MoSi_2_, TlI (B33, *Cmcm*), Cu_3_Ti, CsCl, NH_4_Pb_2_Br_5_, Mn_2_AlB_2_, Cr_5_Si_3_B, and TiNiSi). However, many questions regarding the peculiarities of the chemical processes remain open. For example, we observed Na-containing compounds upon heating Dy in NaCl—*hP*18 Na_4_Cl_5_ ^54^ at 95 GPa (Tables S1 and S9) and *oI*32 DyNa_2_Cl_5_ at 107(3) GPa (Tables S1 and S4)—in a single experiment. What happened with Na in other experiments is unclear. We can speculate that Na-containing compounds may be amorphous or decompose upon temperature quenching, but resolving this issue requires further *in situ* HPHT experiments.

The design of an experiment, especially the position of a sample in the pressure chamber, may affect its outcome, and under similar PT conditions, the predominant reaction products may be different. However, a definite conclusion, whether direct contact of metal with the diamond (Figure 1a) facilitates the synthesis of carbides, whereas isolation of metal from anvils by the pressure medium (NaCl) (Figure 1b) stimulates the synthesis of Cl-containing compounds, cannot yet be made. In all experiments in this work and our previous studies,^63,64^ a piece of metal was placed directly on the diamond culet (Figure 1a); however, at 19–70 GPa in refs 63 and 64, the primary products were carbides, such as γ-DyC_2_, Dy_5_C_9_, Dy_2_C_3_, γ-Dy_4_C_5_, Dy_4_C_3_, and Dy_3_C_2_, whereas in the present work at similar PT conditions, both chlorides and chloride carbides were synthesized. In ref. 4, at 40 GPa with the experimental design according to Figure 1b, the main products were DyCl and the chloride carbide Dy_2_ClC.^4^

It would be interesting to see whether there is a correlation between the atomic radii of the REEs and their reactivity with NaCl (Figure S1). Sc pressurized and heated in NaCl at 64 GPa formed only Sc_4_C_3_ carbide known at ambient conditions.^65^ In contrast, heating Sm in NaCl at 62 GPa resulted in the synthesis of both novel carbides and a chloride carbide, Sm_2_ClC_2_ (Table S1). Dy seems to be the most reactive compared to all other studied REEs, but it is not clear if this relates to its enhanced reactivity, more studies conducted, or the particular design of the experiments. More studies are needed to clarify this point.

The formation of zigzag (*trans*-polyacetylene-like) carbon chains was previously suggested for the Li–C and Ca–C systems^66–68^ but has never been observed. In this study, such chains were identified for the first time in a series of isostructural lanthanide (Dy, Gd, and Sm) chloride carbides, *oC*10 Dy_2_ClC_2_, Gd_2_ClC_2_, and Sm_2_ClC_2_, with an *oC*10 Mn_2_AlB_2_-type structure (Figure 4a). Interestingly, the structure of these chloride carbides is very similar to that of *tI*6 La_2_Cl (Figure 2a). Both the former and the latter are built of layers of edge-sharing unoccupied metal–chlorine Me_2_Cl_4_ octahedra stacking one upon another. However, in the chloride carbides, these layers alternate with the layers of zigzag carbon chains (Figure 4a). Notably, there is another polymorph of a rare-earth metal chloride with the same stoichiometry, *tI*12 Y_2_Cl^4^, which adopts a different structure.

Considering the interatomic distances in *oC*10 Dy_2_ClC_2_ at 76(3) GPa, metal atoms are coordinated by four nearest chlorine (*d*(Dy–Cl) = 2.6412(8) Å) and six nearest carbon atoms (*d*(Dy–C) = 2.307(7) or 2.365(11) Å) (Figure 4a, right)—the latter being shorter than *d*(Dy–C) in γ-DyC_2_ (*d*(Dy–C) ∼ 2.43 Å) and Dy_4_C_3_ (*d*(Dy–C) ∼ 2.39 Å) at similar pressures (according to the EoSs in ref 64). It seems common that the presence of chlorine anions in the structure results in a shortening of the metal–carbon distances in chloride carbides. For example, in the aforementioned *hP*18 Dy_5_Cl_3_C *d*(Dy–C) = 2.1444(3) Å, which is also shorter than in Dy_4_C_3_ (*d*(Dy–C) ∼ 2.26 Å—as expected based on the EoS^64^). This observation can be attributed to charge redistribution in the synthesized ternary metal–chlorine–carbon compounds. The highly electronegative chlorine withdraws electron density from the metal atoms, increasing their positive charge and strengthening their interaction with carbon.

## Conclusion

To summarize, the chemical reactions between NaCl and La, Sm, Gd, or Dy, observed in this work upon laser heating of the metals to 2500–2800 K at 39–127 GPa in DACs, resulted in the synthesis of previously unknown chlorides (La_2_Cl, LaCl, LaCl_3_, and DyCl), chloride carbides (Sm_2_ClC_2_, Gd_2_ClC_2_, Dy_2_ClC_2_, Sm_19_ClC_18_, Gd_19_ClC_18_, Dy_5_Cl_3_C), an oxide chloride (DyOCl), and a ternary chloride (DyNa_2_Cl_5_). The latter is the first observation of a ternary Na-containing compound as a product of the HPHT reaction with NaCl. For the first time, zigzag (*trans*-polyacetylene-like) carbon chains have been identified in *oC*10 Mn_2_AlB_2_-type lanthanide chloride carbides. Additionally, it was demonstrated that the presence of chlorine atoms shortens metal–carbon distances in lanthanide chloride carbides compared to previously reported carbides.^64^ These findings enhance our understanding of chemical bonding in ternary rare-earth compounds under high pressure, paving the way for further studies of their crystal chemistry under extreme conditions.

Our methodology for the synthesis of novel chlorides and chloride carbides by the direct reaction of metals with halides at high pressure and temperature in laser-heated DACs results in producing phase mixtures. Although the individual phases cannot yet be separated, our approach allows investigating “pressure–temperature-phase-structure” space and significantly expands our knowledge of materials that can be synthesized under certain PT conditions.

## Supplementary Material



## Notes

Summary of the performed DAC laser heating experiments and crystallographic information (PDF)
