# Supplementary material for: High-Pressure Synthesis and Structural Studies of La, Sm, Gd, and Dy Chlorides and Chloride Carbides
Source: ACS Omega. 2026 Jan 9;11(3):4280–9. doi: 10.1021/acsomega.5c09373 (PMC12854611; doi:10.1021/acsomega.5c09373)
Supplement: Supplementary file 1 [file ao5c09373_si_001.pdf]

Supporting Information

High-pressure synthesis and structural studies of La, Sm, Gd and Dy chlorides and chloride carbides

Fariia Iasmin Akbar[1,2*], Alena Aslandukova[1,2], Andrey Aslandukov[1,3], Yuqing Yin[2,4], Elena Bykova[5], Maxim Bykov[1], Dominique Laniel[6], Pavel Milkin[7], Timofey Fedotenko[8], Jonathan Wright[9], Anna Pakhomova[9], Gaston Garbarino[9], Mohamed Mezouar[9], Michael Hanfland[9], Natalia Dubrovinskaia[3], and Leonid Dubrovinsky[2*]

[1] Institute of Inorganic and Analytical Chemistry, Goethe University Frankfurt, Max-von-Laue-Straße 7, 60438 Frankfurt, Germany

[2] Bavarian Research Institute of Experimental Geochemistry and Geophysics (BGI), University of Bayreuth, Universitätsstraße 30, 95440 Bayreuth, Germany

[3] Material Physics and Technology at Extreme Conditions, Bavarian Research Institute of Experimental Geochemistry and Geophysics (BGI), University of Bayreuth, Universitätsstraße 30, 95440 Bayreuth, Germany

[4] Department of Physics, Chemistry and Biology (IFM), Linköping University, SE-581 83, Linköping, Sweden

[5] Institute of Geosciences, Goethe University Frankfurt, Altenhöferallee 1, 60438 Frankfurt am Main, Germany

[6] Centre for Science at Extreme Conditions and School of Physics and Astronomy, University of Edinburgh, EH9 3FD Edinburgh, United Kingdom

[7] Faculty of Engineering Sciences, University of Bayreuth, Ludwig Thoma Str. 36A, Bayreuth 95447, Germany

[8] Deutsches Elektronen-Synchrotron DESY, Notkestrasse 85, 22607 Hamburg, Germany

[9] European Synchrotron Radiation Facility, CS 40220, 38043 Grenoble Cedex 9, France

***Corresponding authors:** Fariia Iasmin Akbar akbar@chemie.uni-frankfurt.de; Leonid Dubrovinsky Leonid.Dubrovinsky@uni-bayreuth.de

Table S1. Summary of the high-pressure high-temperature experiments performed in diamond anvil cells. Novel chlorides, chloride carbides, and an oxychloride synthesized in the course of this work are highlighted in **bold**; previously known compounds found among the reaction products are *italicized*. Rare-earth carbides, noted as 'REE-C compounds' and marked with asterisks**, are not a subject of the current paper and will be presented in a separate publication.

DAC number	Starting materials	Culet size (μm)	Pressure (GPa)	Temperature (K, ±200)	Reaction products
LA01*	Dy in NaCl	250	19(1)	2500	Dy_4C_3 [1], Dy_2C_3 [1]
LA04*	Dy in NaCl	250	55(1)	2500	Dy_4C_3 [1], Dy_3C_2 [1]
LA05	Dy in NaCl	120	76(3)	2500	**Dy_2ClC_2**, **DyOCl**
			95(3)	2800	*Na_4Cl_5* [2], **DyCl**, Dy_4C_3 [3]
			107(3)	2500	**$DyNa_2Cl_5$**, **DyOCl**
LA08	Dy in NaCl	80	122(3)	2800	**DyCl**, **Dy_5Cl_3C**
LA09*	Sc in NaCl	250	64(1)	2500	Sc_4C_3 [4,5]**
LA11	Gd in NaCl	250	45(2)	2500	**$Gd_{19}ClC_{18}$**, Gd-C compounds**
			58(2)	2500	**Gd_2ClC_2**, Gd-C compounds**
LA14	Sm in NaCl	250	39(2)	2500	**$Sm_{19}ClC_{18}$**, Sm-C compound**
			62(2)	2500	**Sm_2ClC_2**, Sm-C compounds**
LA16	La in NaCl	120	80(2)	2500	**La_2Cl**, **LaCl**, **$LaCl_3$**
LA28	Sm in NaCl	120	127(2)	2500	**Sm_2ClC_2**, Sm-C compound**
ALA33*	Y in NaCl	120	68(3)	2500	Y-C compound**
			94(3)	2500	Y-C compound**
			124(3)	2500	Y-C compound**

* No binary or ternary chlorine-containing compounds were detected.

** REE-C compounds synthesized in this work will be described elsewhere.

Table S2. Structure refinement details and crystallographic data of *tI*6 La_2Cl, *oC*8 LaCl, and *oP*8 $LaCl_3$ at 80(2) GPa. The full crystallographic datasets were deposited to the CCDC under the deposition numbers 2477211 (La_2Cl at 81(2) GPa), 2477212 (LaCl at 80(2) GPa), 2477215 ($LaCl_3$ at 81(2) GPa).

Chemical formula	La_2Cl		LaCl		$LaCl_3$	
Pressure (GPa)	81(2)		80(2)		81(2)	
Crystal data						
M_r	313.27		174.36		245.26	
ρ (g/cm^3)	9.067		8.853		7.339	
Radiation type	X-ray, λ = 0.2904 Å					
Space group	*I*4/*mmm*		*Cmcm*		*Pmmn*	
a (Å)	3.0029(4)		3.2669(12)		4.434(2)	
b (Å)	3.0029(4)		9.789(7)		5.349(8)	
c (Å)	12.725(7)		4.0906(19)		4.679(2)	
V (Å^3)	114.74(7)		130.82(12)		110.98(19)	
Z	2		4		2	
CN of La1	4		7		12	
La1-Cl in first coordination sphere (Å)	2.7579(12)		2.6548(15)-2.728(8)		2.675(5)-2.728(3)	
Atom / Wyck. site/ Fractional atomic coordinates (*x*; *y*; *z*) and equivalent isotropic (or isotropic) ADPs (Å^2)	La1/ 4*e*	0 0 0.36170(12)	La1/ 4*c*	0 0.36668(19) 0.25	La1/ 2*a*	0.25 0.25 0.17383(19)
		U_{eq} = 0.0070(3)		U_{eq} = 0.0086(5)		U_{eq} = 0.0096(3)
	Cl1/ 2*a*	0 0 0	Cl1/ 4*c*	0 0.0880(7) 0.25	Cl1/ 4*e*	0.25 0.0007(7) 0.6720(5)
						U_{eq} = 0.0083(7)
		U_{eq} = 0.0054(13)		U_{iso} = 0.0079(10)	Cl2/ 2*b*	0.25 0.75 0.1660(8)
						U_{eq} = 0.0087(9)

Data collection			
No. of measured, independent and observed [I > 2σ(I)] reflections	271/95/75	162/75/66	455/219/184
R_{int}	3.31%	2.57%	2.86%
Refinement			
R_1	3.48%	3.04%	4.57%
wR_2	7.62%	7.02%	11.24%
GOF	1.001	1.256	1.106
No. of reflections/No. of parameters	95/6	75/7	219/15

Table S3. Structure refinement details and crystallographic data of *cP*2 DyCl at 95(3) and 121(3) GPa. The full crystallographic datasets were deposited to the CCDC under the deposition numbers 2477205 (DyCl at 95(3) GPa), 2477207 (DyCl at 121(3) GPa).

Chemical formula		DyCl	
Pressure (GPa)		95(3)	121(3)
Crystal data			
M_r		197.95	
ρ (g/cm^3)		13.234	14.081
Radiation type		X-ray, λ = 0.2844 Å	
Space group		*Pm*-3*m*	
a (Å)		2.9177(7)	2.85799(15)
V (Å^3)		24.839(18)	23.344(4)
Z		1	
CN of Dy1		8	
Dy1-Cl in first coordination sphere (Å)		2.5268(7)	2.47509(14)
Atom / Wyck. site/ Fractional atomic coordinates (*x*; *y*; *z*) and equivalent isotropic ADPs (Å^2)	Dy1/1*b*	0.5 0.5 0.5	
		U_{eq} = 0.0067(2)	U_{eq} = 0.0063(6)
	Cl1/1*a*	0 0 0	
		U_{eq} = 0.0084(8)	U_{eq} = 0.0060(2)
Data collection			
No. of measured, independent and observed [I > 2σ(I)] reflections		114/35/35	141/37/37
R_{int}		2.91%	2.79%
Refinement			
R_1		2.54%	2.31%
wR_2		4.27%	5.83%
GOF		1.263	1.180
No. of reflections/No. of parameters		35/3	37/3

Table S4. Structure refinement details and crystallographic data of *oI*32 $DyNa_2Cl_5$ at 107(3) GPa. The full crystallographic dataset was deposited to the CCDC under the deposition number 2477217.

Chemical formula		$DyNa_2Cl_5$
Pressure (GPa)		107(3)
Crystal data		
M_r		385.73
ρ (g/cm^3)		7.153
Radiation type		X-ray, λ = 0.2846 Å
Space group		*I*4/*mcm*
a (Å)		5.9250(14)
c (Å)		10.202(10)
V (Å^3)		358.2(4)
Z		4
CN of Dy1		10
CN of Na1		8
Dy1-Cl in first coordination sphere (Å)		2.4635(9), 2.550(3)
Na1-Cl in first coordination sphere (Å)		2.2792(15)-2.3208(15)
Atom / Wyck. site/ Fractional atomic coordinates (*x*; *y*; *z*) and equivalent isotropic ADPs (Å^2)	Dy1/4*a*	0 0 0.25
		U_{eq} = 0.00590(8)
	Cl1/16*l*	0.15731(7) 0.65731(7) 0.14826(8)
		U_{eq} = 0.00634(14)
	Cl2/4*c*	0 0 0
		U_{eq} = 0.0054(3)
	Na1/8*h*	0.63922(19) 0.13922(19) 0
		U_{eq} = 0.0046(3)
Data collection		
No. of measured, independent and observed [I > 2σ(I)] reflections		1085/300/258
R_{int}		2.02%
Refinement		
R_1		2.13%
wR_2		5.15%

GOF	1.045
No. of reflections/No. of parameters	300/15

Table S5. Structure refinement details and crystallographic data of *oC*10 Sm_2ClC_2 at 62(2) GPa, *oC*10 Gd_2ClC_2 at 58(2) GPa, and *oC*10 Dy_2ClC_2 at 76(3) GPa. The full crystallographic datasets were deposited to the CCDC under the deposition numbers 2477213 (Sm_2ClC_2 at 62(2) GPa), 2477208 (Sm_2ClC_2 at 127(2) GPa), 2477214 (Gd_2ClC_2 at 58(2) GPa), 2477209 (Dy_2ClC_2 at 76(3) GPa).

Chemical formula		Sm_2ClC_2		Gd_2ClC_2	Dy_2ClC_2
Pressure (GPa)		62(2)	127(2)	58(2)	76(3)
Crystal data					
M_r		360.17		373.97	384.47
ρ (g/cm^3)		10.670	12.408	11.082	12.348
Radiation type		X-ray, λ = 0.3738 Å		X-ray, λ = 0.4100 Å	X-ray, λ = 0.2846 Å
Space group		*Cmmm*			
a (Å)		2.7866(5)	2.6663(3)	2.8074(9)	2.734(2)
b (Å)		13.105(5)	12.4435(14)	13.029(3)	12.656(2)
c (Å)		3.0697(5)	2.906(3)	3.0640(3)	2.9884(8)
V (Å^3)		112.10(5)	96.41(8)	112.07(5)	103.41(9)
Z		2			
CN of REE1		10			
REE1-Cl in first coordination sphere (Å)		2.7159(12)	2.5534(11)	2.7255(12)	2.6412(8)
REE1-C in first coordination sphere (Å)		2.39(4), 2.40(2)	2.289(12), 2.326(18)	2.32(2), 2.423(14)	2.307(7), 2.365(11)
C-C distance in the chain (Å)		1.53(4)	1.49(2)	1.488(18)	1.549(14)
Atom / Wyck. site/ Fractional atomic coordinates (*x*; *y*; *z*) and equivalent isotropic (or	REE1/4*j*	0 0.36610(13) 0.5	0 0.36964(8) 0.5	0 0.36462(13) 0.5	0 0.36603(5) 0.5
		U_{eq} = 0.0114(5)	U_{eq} = 0.0060(7)	U_{eq} = 0.0101(4)	U_{eq} = 0.0060(3)
	Cl1/2*a*	0 0 0	0 0 0	0 0 0	0 0 0
		U_{eq} = 0.0094(16)	U_{iso} = 0.0040(11)	U_{eq} = 0.0087(18)	U_{iso} = 0.0076(9)
	C1/4*i*	0 0.226(3) 0	0 0.2237(18) 0	0 0.231(2) 0	0 0.2212(11) 0

isotropic) ADPs (Å^2)	U_{eq} = 0.015(7)	U_{iso} = 0.010(4)	U_{iso} = 0.008(5)	U_{iso} = 0.007(3)
Data collection				
No. of measured, independent and observed [I > 2σ(I)] reflections	154/96/83	162/83/74	137/77/60	268/104/96
R_{int}	2.24%	5.14%	4.91%	3.35%
Refinement				
R_1	4.85%	3.92%	3.91%	2.98%
wR_2	12.46%	8.10%	8.60%	6.68%
GOF	1.068	1.033	0.950	1.084
No. of reflections/No. of parameters	96/12	83/8	77/10	104/8

Table S6. Structure refinement details and crystallographic data of *mP*76 $Sm_{19}Cl_{0.81}C_{18}$ at 39(2) GPa and *mP*76 $Gd_{19}ClC_{18}$ 45(2) GPa. The full crystallographic datasets were deposited to the CCDC under the deposition numbers 2477220 ($Sm_{19}Cl_{0.81}C_{18}$ at 39(2) GPa), 2477216 ($Gd_{19}ClC_{18}$ at 45(2) GPa).

Chemical formula	$Sm_{19}Cl_{0.81}C_{18}$	$Gd_{19}ClC_{18}$
Pressure (GPa)	39(2)	45(2)
Crystal data		
M_r	3101.54	3239.38
ρ (g/cm^3)	10.379	11.294
Radiation type	X-ray, λ = 0.3738 Å	X-ray, λ = 0.2846 Å
Space group	$P2_1/m$	
a (Å)	7.3574(9)	7.3224(14)
b (Å)	18.6441(19)	18.091(3)
c (Å)	7.943(2)	7.860(2)
β (°)	114.37(3)	113.82(3)
V (Å^3)	992.5(4)	952.5(4)
Z	2	
CN of REE1	7	7
CN of REE2	8	8
CN of REE3	7	7
CN of REE4	7	7
CN of REE5	7	7
CN of REE6	7	7
CN of REE7	7	7
CN of REE8	7	7
CN of REE9	7	7
CN of REE10	6	6
CN of REE11	6	6
REE1-C in first coordination sphere (Å)	2.47(4)-2.567(19)	2.40(3)-2.54(3)
REE2-C/Cl in first coordination sphere (Å)	2.40(3)-2.75(4) / 3.040(6)	2.37(4)-2.74(4) / 2.953(5)

REE3-C in first coordination sphere (Å)		2.38(3)-2.759(16)	2.36(3)-2.65(3)
REE4-C in first coordination sphere (Å)		2.47(3)-2.64(2)	2.43(3)-2.63(2)
REE5-C/Cl in first coordination sphere (Å)		2.44(4)-2.587(15) / 2.881(14)	2.37(4)-2.63(3) / 2.820(11)
REE6-C/Cl in first coordination sphere (Å)		2.42(3)-2.55(3) / 2.710(9)	2.33(3)-2.55(3) / 2.695(9)
REE7-C in first coordination sphere (Å)		2.38(3)-2.87(3)	2.36(3)-2.83(3)
REE8-C/Cl in first coordination sphere (Å)		2.29(2)-2.77(5) / 2.612(10)	2.28(3)-2.55(4) / 2.593(11)
REE9-C/Cl in first coordination sphere (Å)		2.28(2)-2.557(19) / 2.803(17)	2.30(3)-2.80(3) / 2.778(13)
REE10-C in first coordination sphere (Å)		2.29(3)-2.73(3)	2.26(4)-2.62(4)
REE11-C/Cl in first coordination sphere (Å)		2.532(15)-2.92(4) / 2.814(12)	2.40(3)-2.70(3) / 2.768(10)
REE12-C in first coordination sphere (Å)		2.453(15)	-
Atom / Wyck. site/ Fractional atomic coordinates (*x*; *y*; *z*) and equivalent isotropic (or isotropic) ADPs (Å^2)	REE1/4*f*	0.29495(12) 0.00642(4) 0.66243(19)	20360(16) 0.00790(7) 0.84009(19)
		U_{eq} = 0.0099(3)	U_{eq} = 0.0128(3)
	REE2/4*f*	0.30683(12) 0.60967(5) 0.36744(18)	0.19163(16) 0.61037(7) 0.13191(19)
		U_{eq} = 0.0101(3)	U_{eq} = 0.0120(2)
	REE3/4*f*	0.29576(12) 0.00286(4) 0.06860(18)	0.20490(16) 0.00218(7) 0.43376(19)
		U_{eq} = 0.0091(3)	U_{eq} = 0.0121(2)
	REE4/4*f*	0.03476(12) 0.02118(5) 0.26029(18)	0.46391(16) 0.02108(7) 0.23854(18)
		U_{eq} = 0.0088(3)	U_{eq} = 0.0116(2)

	REE5/4*f*	0.79002(12) 0.15755(4) 0.06719(18)	0.70423(16) 0.15847(7) 0.43031(19)
		U_{eq} = 0.0098(3)	U_{eq} = 0.0130(3)
	REE6/4*f*	0.15629(13) 0.15580(4) 0.81482(19)	0.34239(16) 0.15524(7) 0.68592(18)
		U_{eq} = 0.0102(3)	U_{eq} = 0.0122(2)
	REE7/4*f*	0.21173(13) 0.14980(5) 0.44796(19)	0.28903(18) 0.14928(8) 0.0527(2)
		U_{eq} = 0.0118(3)	U_{eq} = 0.0169(3)
	REE8/2*e*	0.50034(19) 0.25 0.7564(3)	0.0022(2) 0.25 0.7501(3)
		U_{eq} = 0.0126(4)	U_{eq} = 0.0171(4)
	REE9/2*e*	0.82076(18) 0.25 0.4485(3)	0.6815(2) 0.25 0.0545(3)
		U_{eq} = 0.0118(4)	U_{eq} = 0.0149(3)
	REE10/4*f*	0.48639(13) 0.13316(6) 0.2248(2)	0.01470(19) 0.13167(9) 0.2734(2)
		U_{eq} = 0.0158(3)	U_{eq} = 0.0220(3)
	REE11/2*e*	0.2005(2) 0.25 0.1702(4)	0.3054(3) 0.25 0.3397(4)
		U_{eq} = 0.0130(5)	U_{eq} = 0.0276(5)
	REE12/2*e*	0.1117(12) 0.25 0.0888(17)	-
		U_{eq} = 0.0130(5)	
	Cl1/2*e*	0.8782(12) 0.25 0.820(2)	0.6252(14) 0.25 0.6831(15)
		U_{eq} = 0.027(3)	U_{eq} = 0.0234(18)
	C1/4*f*	0.018(2) 0.5796(9) 0.048(4)	0.522(4) 0.0794(15) 0.546(4)
		U_{iso} = 0.013(3)	U_{iso} = 0.020(5)

	C2/2*e*	0.577(4) 0.25 0.128(6)	0.978(3) 0.25 0.417(4)
		U_{iso} = 0.016(4)	U_{iso} = 0.003(4)
	C3/4*f*	0.518(2) 0.6199(9) 0.203(4)	0.015(3) 0.1171(13) 0.702(4)
		U_{iso} = 0.013(3)	U_{iso} = 0.014(4)
	C4/4*f*	0.017(2) 0.5773(8) 0.461(4)	0.478(3) 0.5798(13) 0.038(4)
		U_{iso} = 0.012(3)	U_{iso} = 0.014(4)
	C5/4*f*	0.122(2) 0.1185(8) 0.096(3)	0.374(3) 0.1203(12) 0.398(4)
		U_{iso} = 0.009(2)	U_{iso} = 0.011(4)
	C6/2*e*	0.159(3) 0.2500 0.607(5)	0.342(4) 0.25 0.887(4)
		U_{iso} = 0.012(4)	U_{iso} = 0.006(5)
	C7/4*f*	0.504(3) 0.2106(11) 0.460(5)	0.006(4) 0.2131(15) 0.052(4)
		U_{iso} = 0.020(4)	U_{iso} = 0.022(5)
	C8/4*f*	0.540(2) 0.5857(8) 0.050(3)	0.037(3) 0.0852(12) 0.552(4)
		U_{iso} = 0.008(2)	U_{iso} = 0.012(4)
	C9/4*f*	0.140(2) 0.6182(9) 0.619(4)	0.644(3) 0.1173(12) 0.125(4)
		U_{iso} = 0.013(3)	U_{iso} = 0.012(4)
	C10/4*f*	0.428(3) 0.0246(9) 0.427(4)	0.072(3) 0.0237(15) 0.079(4)
		U_{iso} = 0.016(3)	U_{iso} = 0.019(5)
Occupancy	REE11/2*e*	0.811(4)	1
	REE12/2*e*	0.189(4)	-

	Cl1/2*e*	0.811(4)	1
Data collection			
No. of measured, independent and observed [I > 2σ(I)] reflections		3248/1932/1600	2733/1564/1272
R_{int}		2.36%	2.37%
Refinement			
R_1		4.66%	4.54%
wR_2		13.31%	10.89%
GOF		1.067	1.035
No. of reflections/No. of parameters		1932/138	1564/135

Table S7. Structure refinement details and crystallographic data of *hP*18 Dy_5Cl_3C at 122(3) GPa. The full crystallographic dataset was deposited to the CCDC under the deposition number 2477210.

Chemical formula		Dy_5Cl_3C
Pressure (GPa)		122(3)
Crystal data		
M_r		930.86
ρ (g/cm^3)		15.230
Radiation type		X-ray, λ = 0.2844 Å
Space group		$P6_3/mcm$
a (Å)		6.9605(4)
c (Å)		4.8377(12)
V (Å^3)		202.98(6)
Z		2
CN of Dy1		6
CN of Dy2		7
Dy1-Cl in first coordination sphere (Å)		2.4666(4)
Dy2-Cl in first coordination sphere (Å)		2.3880(7)-2.5969(7)
Dy2-C in first coordination sphere (Å)		2.1444(3)
Atom / Wyck. site/ Fractional atomic coordinates (x; y; z) and equivalent isotropic ADPs (Å^2)	Dy1/4*d*	0.3333 0.6667 0
		U_{eq} = 0.00469(7)
	Dy2/6*g*	0.25441(2) 0 0.25
		U_{eq} = 0.00500(6)
	Cl/6*g*	0.60981(12) 0 0.25
		U_{eq} = 0.0049(2)
	C/2*b*	0 0 0
		U_{eq} = 0.009(2)
Data collection		
No. of measured, independent and observed [I > 2σ(I)] reflections		1336/276/238
R_{int}		2.86%
Refinement		
R_1		1.53%

wR_2	2.62%
GOF	1.080
No. of reflections/No. of parameters	276/13

Table S8. Structure refinement details and crystallographic data of *oP*12 DyOCl at 76(3) and 108(3) GPa. The full crystallographic datasets were deposited to the CCDC under the deposition numbers 2477206 (DyOCl at 76(3) GPa), 2477219 (DyOCl at 108(3) GPa).

<table>
<tr><td colspan="2">Chemical formula</td><td colspan="2">DyOCl</td></tr>
<tr><td colspan="2">Pressure (GPa)</td><td>76(3)</td><td>108(3)</td></tr>
<tr><td colspan="4">Crystal data</td></tr>
<tr><td colspan="2">M_r</td><td colspan="2">213.95</td></tr>
<tr><td colspan="2">ρ (g/cm^3)</td><td>10.512</td><td>11.296</td></tr>
<tr><td colspan="2">Radiation type</td><td colspan="2">X-ray, λ = 0.2846 Å</td></tr>
<tr><td colspan="2">Space group</td><td colspan="2">Pnma</td></tr>
<tr><td colspan="2">a (Å)</td><td>5.873(3)</td><td>5.7493(10)</td></tr>
<tr><td colspan="2">b (Å)</td><td>3.619(4)</td><td>3.533(2)</td></tr>
<tr><td colspan="2">c (Å)</td><td>6.360(3)</td><td>6.1939(16)</td></tr>
<tr><td colspan="2">V (Å^3)</td><td>135.19(19)</td><td>125.81(8)</td></tr>
<tr><td colspan="2">Z</td><td colspan="2">4</td></tr>
<tr><td colspan="2">CN of Dy1</td><td colspan="2">10</td></tr>
<tr><td colspan="2">Dy1-Cl/O in first coordination sphere (Å)</td><td>2.481(4)-3.017(4) / 2.136(11)-2.189(10)</td><td>2.4280(12)-2.9279(13) / 2.105(4)-2.112(4)</td></tr>
<tr><td rowspan="6">Atom / Wyck. site/ Fractional atomic coordinates (x; y; z) and equivalent isotropic (or isotropic) ADPs (Å^2)</td><td rowspan="2">Dy1/4c</td><td>0.29862(15) 0.25 0.39232(11)</td><td>0.29759(4) 0.25 0.39290(4)</td></tr>
<tr><td>U_{eq} = 0.0101(3)</td><td>U_{eq} = 0.00569(8)</td></tr>
<tr><td rowspan="2">Cl1/4c</td><td>0.0057(6) 0.25 0.6735(5)</td><td>0.00457(17) 0.25 0.67519(18)</td></tr>
<tr><td>U_{eq} = 0.0079(11)</td><td>U_{eq} = 0.0054(3)</td></tr>
<tr><td rowspan="2">O1/4c</td><td>0.1603(18) 0.25 0.0727(14)</td><td>0.1622(6) 0.25 0.0760(5)</td></tr>
<tr><td>U_{iso} = 0.007(2)</td><td>U_{eq} = 0.0042(8)</td></tr>
<tr><td colspan="4">Data collection</td></tr>
<tr><td colspan="2">No. of measured, independent and observed [I > 2σ(I)] reflections</td><td>300/132/107</td><td>859/399/328</td></tr>
<tr><td colspan="2">R_{int}</td><td>2.20%</td><td>1.79%</td></tr>
<tr><td colspan="4">Refinement</td></tr>
<tr><td colspan="2">R_1</td><td>2.48%</td><td>2.06%</td></tr>
</table>

wR_2	5.79%	5.15%
GOF	1.026	1.030
No. of reflections/No. of parameters	132/16	399/19

Table S9. Structure refinement details and crystallographic data of *hP*18 Na_4Cl_5 at 95(3). The full crystallographic datasets were deposited to the CCDC under the deposition numbers 2477218.

Chemical formula		Na_4Cl_5
Pressure (GPa)		95(3)
Crystal data		
M_r		269.21
ρ (g/cm^3)		4.726
Radiation type		X-ray, λ = 0.2843 Å
Space group		$P6_3/mcm$
a (Å)		6.9295(11)
c (Å)		4.5497(10)
V (Å^3)		189.20(7)
Z		2
CN of Na1		6
CN of Na2		9
Na1-Cl in first coordination sphere (Å)		2.2392(9)
Na1-Cl in first coordination sphere (Å)		2.356(3)-2.4430(8)
Atom / Wyck. site/ Fractional atomic coordinates (x; y; z) and equivalent isotropic ADPs (Å^2)	Na1/2*b*	0 0 0
		U_{eq} = 0.0085(6)
	Na2/6*g*	0.6184(3) 0 0.25
		U_{eq} = 0.0087(4)
	Cl1/4*d*	0.3333 0.6667 0
		U_{eq} = 0.0099(2)
	Cl2/6*g*	0.27835(13) 0 0.25
		U_{eq} = 0.00694(18)
Data collection		
No. of measured, independent and observed [I > 2σ(I)] reflections		596/239/158
R_{int}		7.60%
Refinement		

R_1	4.27%
wR_2	7.08%
GOF	0.927
No. of reflections/No. of parameters	239/13

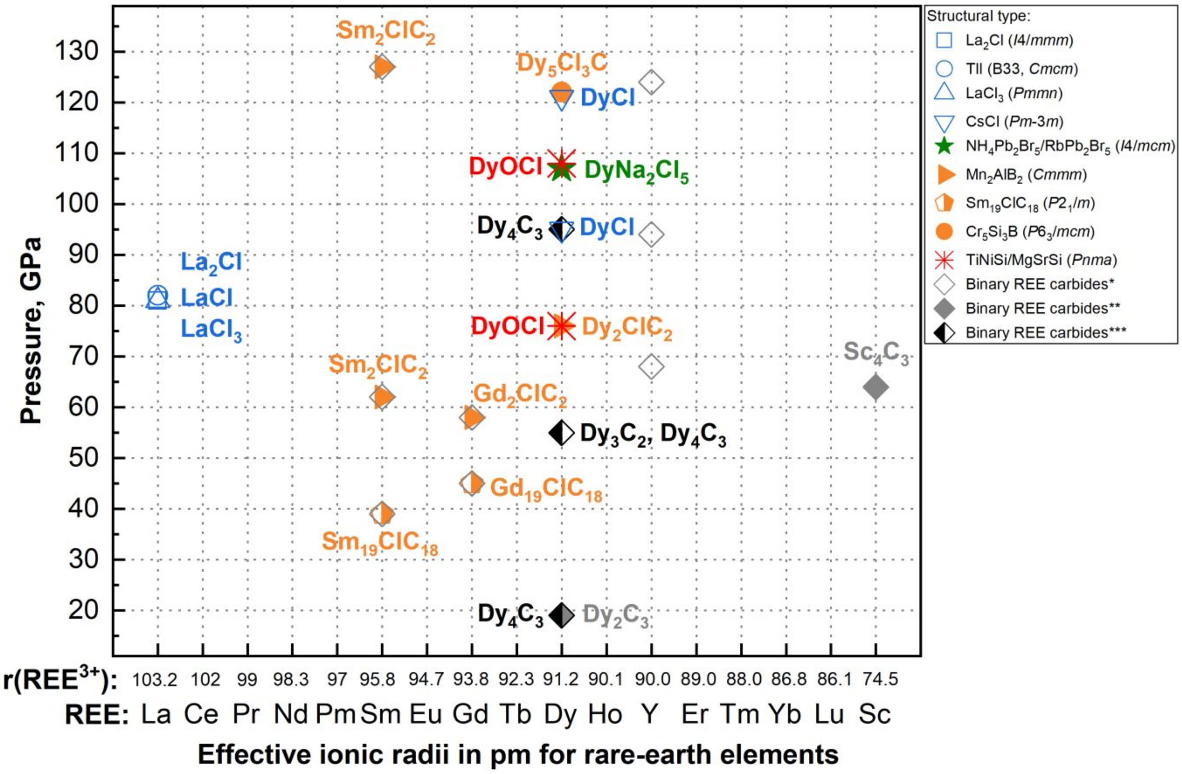

Figure S1. Summary of experiments conducted in this work, which resulted in the synthesis of Cl-containing REE binary and ternary compounds. The products of the high-pressure high-temperature reactions between REE, NaCl, and, in some cases, C from diamond anvils (or O from oxidized samples) are notated by their chemical formulas. Different structure types are shown by symbols of different shapes and colors: binary chlorides – blue, ternary chloride carbides – orange, ternary chloride – green, oxide chloride – red. Grey rhombuses indicate experiments that resulted solely in the synthesis of binary REE carbides, which are not the subject of the present paper. These include: *novel carbides to be presented in a separate publication; **carbides known at ambient conditions; and ***novel carbides recently reported in [1,6]. REE ionic radii r(REE^{3+}) are taken from [7].

References

(1) Akbar, F. I.; Aslandukova, A.; Aslandukov, A.; Yin, Y.; Trybel, F.; Khandarkhaeva, S.; Fedotenko, T.; Laniel, D.; Bykov, M.; Bykova, E.; Dubrovinskaia, N.; Dubrovinsky, L. High-Pressure Synthesis of Dysprosium Carbides. *Front. Chem.* **2023**, *11*, 1–9. https://doi.org/10.3389/fchem.2023.1210081.

(2) Yin, Y.; Aslandukova, A.; Jena, N.; Trybel, F.; Abrikosov, I. A.; Winkler, B.; Khandarkhaeva, S.; Fedotenko, T.; Bykova, E.; Laniel, D.; Bykov, M.; Aslandukov, A.; Akbar, F. I.; Glazyrin, K.; Garbarino, G.; Giacobbe, C.; Bright, E. L.; Jia, Z.; Dubrovinsky, L.; Dubrovinskaia, N. Unraveling the Bonding Complexity of Polyhalogen Anions: High-Pressure Synthesis of Unpredicted Sodium Chlorides Na2Cl3 and Na4Cl5 and Bromide Na4Br5. *JACS Au* **2023**, *3* (6), 1634–1641. https://doi.org/10.1021/jacsau.3c00090.

(3) Akbar, F. I.; Aslandukova, A.; Yin, Y.; Aslandukov, A.; Comboni, D.; Hanfland, M.; Dubrovinskaia, N. High-Pressure Dysprosium Carbides Containing Carbon Dimers, Trimers, Chains, and Ribbons. *Submitt. to Carbon* **2024**.

(4) Krikorian, N. H.; Bowman, A. L.; Krupka, M. C.; Arnold, G. P. The Preparation and Crystal Structure of Sc4C3. *High Temp. Sci.* **1969**, *1*, 360–366.

(5) Krikorian, N. H.; Giorgi, A. L.; Szklarz, E. G.; Krupka, M. C.; Matthias, B. T. Preparation and Superconductivity of Germanium-Stabilized Sc13C10. *J. Less Common Met.* **1969**, *19* (3), 253–257. https://doi.org/10.1016/0022-5088(69)90101-5.

(6) Akbar, F. I.; Aslandukova, A.; Yin, Y.; Aslandukov, A.; Laniel, D.; Bykova, E.; Bykov, M.; Bright, E. L.; Wright, J.; Comboni, D.; Hanfland, M.; Dubrovinskaia, N.; Dubrovinsky, L. High-Pressure Dysprosium Carbides Containing Carbon Dimers, Trimers, Chains, and Ribbons. *Carbon N. Y.* **2024**, *228*, 119374. https://doi.org/10.1016/j.carbon.2024.119374.

(7) Greenwood, N. N.; Earnshaw, A. *Chemistry of the Elements (2nd Edition)*; Butterworth-Heinemann, 1997.
